# Revision of metal-on-metal hip arthroplasty in a tertiary center: A prospective study of 39 hips with between 1 and 4 years of follow-up

**DOI:** 10.3109/17453674.2013.797313

**Published:** 2013-05-31

**Authors:** Alexander D Liddle, Keshtra Satchithananda, Johann Henckel, Shiraz A Sabah, Karuniyan V Vipulendran, Angus Lewis, John A Skinner, Adam W M Mitchell, Alister J Hart

**Affiliations:** ^1^Department of Orthopaedic Surgery; ^2^Department of Radiology, Imperial College, Charing Cross Hospital, London; ^3^Institute of Orthopaedics and Musculoskeletal Science, University College London, Royal National Orthopaedic Hospital, Stanmore, Middlesex, UK.

## Abstract

**Background and purpose:**

Operative findings during revision of metal-on-metal hip arthroplasty (MOMHA) vary widely and can involve massive soft tissue and bone disruption. As a result, planning of theater time and resources is difficult, surgery is challenging, and outcomes are often poor. We describe our experience with revision of MOMHA and provide recommendations for management.

**Patients and methods:**

We present the findings and outcomes of 39 consecutive MOMHAs (in 35 patients) revised in a tertiary unit (median follow-up time 30 (12–54) months). The patients underwent a preoperative work-up including CT, metal artifact reduction sequence (MARS) MRI, and blood metal ion levels.

**Results:**

We determined 5 categories of failure. 8 of 39 hips had conventional failure mechanisms including infection and impingement. Of the other 31 hips, 14 showed synovitis without significant disruption of soft tissue; 6 had a cystic pseudotumor with significant soft tissue disruption; 7 had significant osteolysis; and 4 had a solid pseudotumor. Each category of failure had specific surgical hazards that could be addressed preoperatively. There were 2 reoperations and 1 patient (2 hips) died of an unrelated cause. Median Oxford hip score (OHS) was 37 (9–48); median change (ΔOHS) was 17 (–10 to 41) points. ΔOHS was similar in all groups—except those patients with solid pseudotumors and those revised to metal-on-metal bearings, who fared worse.

**Interpretation:**

Planning in revision MOMHA is aided by knowledge of the different categories of failure to enable choice of appropriate personnel, theater time, and equipment. With this knowledge, satisfactory outcomes can be achieved in revision of metal-on-metal hip arthroplasty.

In patients who present with a painful MOMHA, there is great heterogeneity of clinical presentation, imaging, and operative findings (Browne et al. 2010). This ranges from unexplained hip pain to catastrophic failure. There is a spectrum of findings on MRI, which range from being normal or demonstrating small, cystic pseudotumors—which have a high incidence in well-functioning MOMHA (Hart et al. 2012)—to demonstrating large, solid masses with widespread destruction of muscle and bone. Intraoperative findings show similar heterogeneity.

One commonly reported mechanism of failure of metal-on-metal (MOM) hips is the result of a high rate of material loss (through wear or corrosion, or both), frequently related to suboptimal implant positioning and subsequent edge loading. This has led to the hypothesis that the soft tissue reaction is a dose-dependent reaction to excessive nanoparticle wear debris (Kwon et al. 2009), and can be avoided by accurate implantation to minimize wear. However, other patients show low bearing surface wear rates with low loss of material from all the surfaces of the implants, suggesting that there is a patient-related susceptibility factor. Conventional, stemmed designs have an additional mechanism of failure when compared to hip resurfacings, as a result of loss of material at the trunion-taper interface (Langton et al. 2011).

The variable degree of destruction of muscle and bone makes planning and performance of revision of MOMHA challenging. Series of such revisions have shown a high incidence of dislocation, recurrence, and reoperation (Grammatopoulos et al. 2009), although this appears to improve with experience (De Smet et al. 2011).

The main aim of this study was to report our experience with revision of MOMHA, in a multidisciplinary setting receiving tertiary referrals and employing sophisticated imaging techniques. A secondary aim was to provide recommendations on the basis of this experience for improvement of pre-revision planning and outcomes in patients undergoing revision of a MOM hip.

## Patients and methods

We present preoperative and outcome data on all patients who were revised for a painful MOMHA at our institution between 2007 and 2010. 39 hips in 35 patients (32 females) were revised during the study period. The median age was 61 (25–74) years. For all patients, data in the following categories were collected prospectively: preoperative and postoperative functional scoring, radiological findings, blood analysis (trace metal analysis, renal function tests, inflammatory markers), histology, and microbiology.

During the period covered by this study, our approach to the painful MOM hip has evolved as a result of published research. However, from the outset we adopted a multidisciplinary approach with the assistance of musculoskeletal radiologists, a clinical chemist, a microbiologist with an interest in orthopaedic implants, and a musculoskeletal histopathologist.

We obtained ethical approval for the prospective collection and analysis of our data, which was analyzed at a median follow-up time of 30 (12–54) months. Patients were recruited either locally or as tertiary referrals from surgeons regionally and nationally, and therefore had a range of implant types and surgical approaches. All patients were referred with a diagnosis of unexplained pain attributable to a MOM articulation. Preoperative work-up included metal artifact reduction sequence (MARS) MRI and 3D CT scanning. MARS MRI was performed using a 1.5-Tesla scanner (Magnetom 1.5T; Siemens Medical, Erlangen, Germany). Preoperative CT images were reconstructed in 3 dimensions and anatomical inclination and version was defined with reference to the anterior pelvic plane (Dandachli et al. 2009). This was converted to radiographic values using accepted formulae (Murray 1993). Blood metal ions and functional hip scores were determined preoperatively.

Detailed operative findings were recorded. Patients were followed-up at 6, 12, 26, and 52 weeks with clinical assessment, blood metal ions, plain radiographs, and functional hip scores. Postoperative cross-sectional imaging was reserved for patients who became symptomatic after revision without any clear explanation.

For the purposes of this study, MRI and CT images were reviewed by a consultant radiologist who was blinded regarding the clinical and operative findings, and using a previously published protocol (Sabah et al.. 2011). Specific findings recorded included the presence and appearance of a soft tissue mass, muscle loss, bone death, and loosening/osteolysis. Muscle damage (gluteus maximus, medius, and minimus, piriformis, obturator internus, and obturator externus) was rated from 0 to 3, with 0 being no loss of muscle, 1 being loss of < 30% of the muscle surface area, 2 being 30–70% loss, and 3 being > 70% loss. Patient-reported outcome was recorded using a validated hip score (Murray et al. 2007). On the basis of preoperative and intraoperative findings, and histological examination of tissues collected at operation, failures were classified as being either bearing-related or due to conventional causes such as infection or impingement. After removal, implants underwent wear analysis as described by Matthies et al. (2011).

### Statistics

Descriptive statistics had a non-normal distribution of data and non-parametric tests were used to summarize the data and compare between the categories of failure and implant types. Spearman’s rank correlation was used to determine the presence and strength of any correlation between pre-revision investigation results and clinical outcome. A contingency table was used to determine the sensitivity and specificity of the MRI findings in predicting the operative findings, and the chi-squared test was used to determine the statistical significance of any associations. Wilcoxon’s signed rank test was used to compare clinical outcomes before and after revision. The group was divided into subgroups, which were analyzed using the Mann-Whitney U test and included hips showing high and low degrees of wear, hips with normal and abnormal results of preoperative investigation, and hips with different types of revision procedure. For these tests, effect size was calculated using Hodges-Lehmann estimators, which calculate the differences between each possible pair in each group and give the median of the resultant list of differences. The 95% confidence intervals (CIs) for this statistic are shown alongside the Hodges-Lehmann medians of difference.

This was a consecutive series of hips, so the cohort had some bilateral cases. These bilateral cases have been included in the analyses presented below, to provide as accurate a record as possible of the revision practice of our institution over the time period studied. However, in the analysis of these findings, the statistical tests employed assume independence of all the cases, which may have been compromised by the inclusion of these bilateral cases (Bryant et al. 2006, Park et al. 2010). In order to test the validity of the tests used, the analysis was repeated using a smaller dataset in which the bilateral cases were removed. In the interests of clarity and brevity, all statistics that are shown in the Results section relate to the full dataset, except in instances where the findings of this second analysis diverged from the findings of the complete dataset, where both statistical results are given. Statistical analysis was performed using SPSS version 19.

## Results

Most of the patients were tertiary referrals and this is reflected in the heterogeneity of implant designs revised (Table 1). Most cases were resurfacing prostheses (32/39), and most of these (21/32) were Birmingham Hip Resurfacings (Smith and Nephew Inc., Memphis, TN). Median survivorship after implantation of the primary prosthesis was 47 (11–131) months. Median preoperative OHS was 15 (2–31). Median follow-up time was 30 (12–54) months.

**Table 1.** Preoperative and demographic details

No.	Age	Sex	Prosthesis	Modular	Head	Cup	Incl.	Ver.	Survival (m)	Co	Cr
1	50	F	BHR	Resurfacing	50	56	71	42	31	47	33
2	55	M	BHR	Resurfacing	46	54	29	-34	13	0.9	1.5
3	63	F	ASR	Resurfacing	47	54	55	48	29	30	32
4	65	F	ASR	Resurfacing	40	48	66	28	53	24	21
5	72	F	BHR	Resurfacing	50	56	44	29	59	3.1	2.3
6	65	F	BHR	Resurfacing	42	48	38	9	61	1.1	1.3
7	69	F	BHR	Resurfacing	42	48	56	23	82	1.7	2.7
8	63	F	BHR	Resurfacing	42	50	40	7	95	7.7	9.4
9	46	F	Cormet	Resurfacing	44	50	75	34	18	3.3	0.3
10	65	F	BHR	Resurfacing	42	50	57	26	56	21	11
11	66	M	Cormet	Resurfacing	48	54	60	29	68	12	6.0
12	74	F	BHR	Resurfacing	44	50	34	32	54	1.5	1.0
13	48	F	BHR	Resurfacing	46	54	41	27	45	1.8	6.7
14	48	F	BHR	Resurfacing	48	54	47	26	52	1.8	6.7
15	73	F	Durom	THA	50	56	55	37	38	6.5	8.4
16	35	F	Cormet	Resurfacing	44	50	43	26	11	1.6	8.6
17	57	F	ASR	Resurfacing	53	60	70	43	45	165	117
18	60	M	BHR	Resurfacing	50	56	51	12	43	1.0	2.7
19	67	M	Mitch	THA	44	50	60	-6	13	1.2	0.5
20	64	M	BHR	Resurfacing	50	56	57	43	92	71	36
21	67	F	Biomet	Resurfacing	44	50	49	9	28	9.5	1.0
22	67	F	Biomet	Resurfacing	44	50	38	-5	22	9.5	1.0
23	61	F	BHR	Resurfacing	42	50	64	13	43	2.1	3.1
24	47	F	BHR	Resurfacing	42	50	43	39	65	0.5	0.7
25	74	F	BHR	THA	50	56	37	19	49	8.8	3.0
26	69	M	BHR	Resurfacing	54	60	73	41	63	33	14
27	60	F	BHR	Resurfacing	46	52	36	26	68	1.5	3.5
28	25	F	Cormet	Resurfacing	40	48	47	45	39	70	32
29	61	F	Pinnacle	THA	36	52	68	-3	56	102	37
30	55	F	BHR	Resurfacing	46	50	64	30	72	98	49
31	69	F	ASR	Resurfacing	43	48	55	27	62	11	6.6
32	45	F	ASR	THA	45	50	57	35	38	10	3.5
33	46	F	ASR	THA	45	50			47	10	3.5
34	66	F	BHR	Resurfacing	42	50	66	34	68	105	52
35	61	M	BHR	Resurfacing	46	52	43	22	131	3.8	2.9
36	31	F	Cormet	Resurfacing	40	46	42	24	30	20	6.8
37	58	F	BHR	Resurfacing	46	52	42	20	25	3.1	4.8
38	44	F	Mitch	Resurfacing	52	58	38	38	21	23	13
39	72	F	Taperloc Magnum	THA	44	50			28	9.4	0.5

Incl. and Ver.: inclination and version measured by CT (radiographic values); Co and Cr: whole-blood cobalt and chromium (parts per billion); BHR: Birmingham Hip Resurfacing (Smith and Nephew); ASR: Anatomical Surface Replacement (DePuy Johnson and Johnson).

### Biochemistry (Table 1)

Median preoperative cobalt level was 9.4 (0.5–166) parts per billion (ppb) and median preoperative chromium level was 6.0 (0.26–117) ppb. Levels were below 7 ppb (the level considered acceptable for a unilateral MOMHA, as defined by the UK Medicines and Healthcare Regulatory Agency (MHRA, 2010)) in 17 of the 39 hips for cobalt, and in 24 of the 39 hips for chromium. There was no correlation between preoperative cobalt or chromium levels and preoperative functional scoring or time to revision (Spearman’s correlation, p = 0.12–1.0).

### MRI findings (Table 2)

MRI was performed in 35 of the 39 hips. A mass was present in 21 of 35 hips. Levels of muscle edema or loss varied between patients.

**Table 2.** MRI and intraoperative appearances; revision surgery performed

Hip	MRI mass and class	MRI muscle destruction		Intraoperative appearance	Type	Revision prosthesis and bearing
No.		Abductor	SER			
1	N/A	N/A	N/A	Synovitis, no mass	II	COC
2	Nil	Moderate	Severe	Clear impingement, no mass	I	COC
3	Nil	Moderate	Severe	Synovitis, no mass	II	COC
4	Nil	Moderate	Severe	Synovitis, no mass	II	COC
5	Nil	Moderate	Severe	Osteolysis, acetabular # 2º to minor trauma, no mass	III	COC
6	Nil	Mild	None	Synovitis, no mass	II	MOM
7	N/A	N/A	N/A	Mass, abductors preserved	III	COC
8	2a	None	Mild	Synovitis, no mass	II	COC
9	3	Moderate	Severe	Solid mass, pelvic extension, abductors preserved	V	Revision stem COC
10	2a	Moderate	Moderate	Loose femoral component with osteolysis	IV	COC
11	N/A	N/A	N/A	Synovitis and acetabular loosening. Infection confirmed on histology/microbiology	I	COC
12	N/A	N/A	N/A	Femoral neck fracture, no mass	I	MOM
13	1	Mild	Moderate	Synovitis, no mass	II	COC
14	2a	Mild	Moderate	Synovitis, no mass	II	COC
15	Nil	None	Severe	Impingement, no mass	I	COC
16	Nil	None	None	Synovitis, no mass	II	COC
17	2a	Severe	Moderate	Mass, gluteal dehiscence, bare trochanter	III	COC
18	Nil	Mild	Moderate	Synovitis, no mass	II	COC
19	Nil	Moderate	Moderate	Synovitis, no mass	I	COC
20	2a	Mild	Mild	Massive pelvic osteolysis	IV	Pelvic recon, COC
21	2a	Mild	Moderate	No mass, osteolysis with loose cup	IV	COC
22	2a	Mild	Severe	No mass, osteolysis with loose cup	IV	COC
23	Nil	Moderate	Severe	Loose – loss of fixation with BHR dysplasia cup	I	COC
24	Nil	Moderate	Moderate	Synovitis, no mass	II	COC
25	2a	Severe	Moderate	Complete destruction of SERs, bone death at GT	III	Revision stem, MOP
26	2b	Moderate	Mild	Pelvic osteolysis, no mass	IV	MOP
27	Nil	Mild	Severe	Synovitis, no mass	II	MOP
28	Nil	Nil	Nil	Synovitis, no mass	II	MOP
29	2a	Severe	Moderate	Severe synovitis. No mass	II	MOP
30	2a	Moderate	Moderate	Very large fluid-filled mass, no muscle dehiscence but atrophic	III	MOP
31	Nil	None	Moderate	Synovitis, no mass	II	MOP
32	3	Moderate	Moderate	Solid mass with massive muscle destruction bilaterally	V	Captive cup, MOP
33	3	Moderate	Moderate	Same patient as above	V	Captive cup, MOP
34	3	Mild	Severe	Pelvic osteolysis, no mass	IV	TMT cup, COC
35	Nil	Mild	Moderate	Pelvic osteolysis, no mass	IV	MOP
36	2a	None	Moderate	Infected. No mass	I	COC
37	1	Moderate	Severe	Complete destruction of SERs with mass	III	COC
38	2b	Moderate	Severe	Infected	I	COC
39	3	Severe	Severe	Solid mass with widespread destruction. Symptomatic encasement of sciatic nerve	V	COC

COC = ceramic-on-ceramic; MOM = metal-on-metal; MOP = metal-on-polyethylene; SER = short external rotators

### CT-measured cup position (Figure 1)

37 of the 39 hips had preoperative CT for inclination and version. Median inclination was 51º (29–75) and mean version was 27º (–34 to 48). 10 of these 37 hips had both inclination and version within the range defined as optimal by Grammatopoulos et al. (2010) for hip resurfacing prostheses. Increasing cup inclination and version correlated positively with whole-blood cobalt (p < 0.005 and p = 0.002 for inclination and version, respectively) and chromium (p = 0.009 and p = 0.002). When analysis was restricted to unilateral cases, the correlation between metal ion levels and inclination persisted (Co: p = 0.003; Cr: p = 0.006), but there was no significant correlation with version for either (Co: p = 0.1; Cr: p = 0.07).

**Figure 1.**
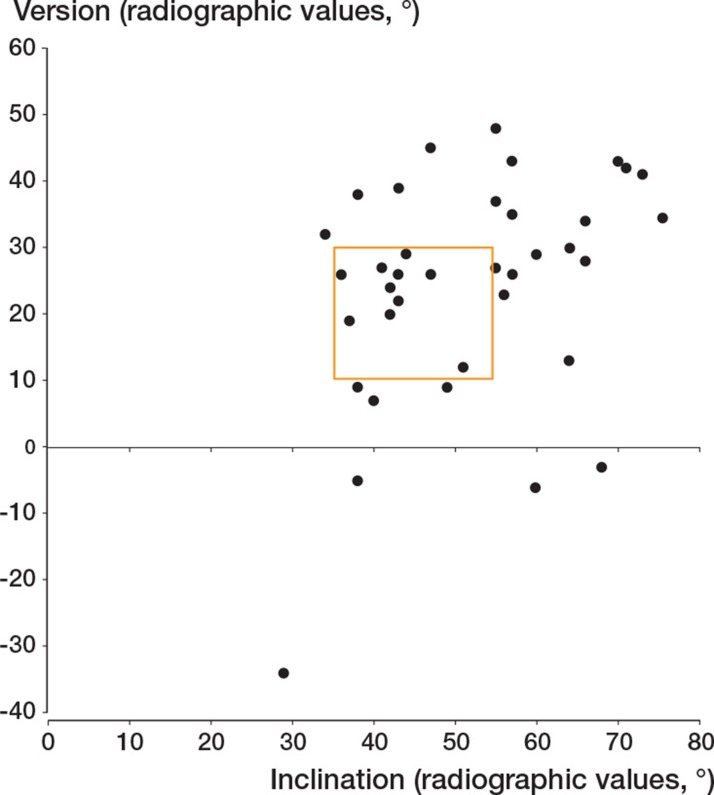
Scatter plot showing inclination and version of the prostheses revised. The box represents optimal position as defined by Grammatopoulos et al. (2009).

### Intraoperative findings and mechanisms of failure (Table 3)

While all hips presented with unexplained pain, 8 of the 39 hips were found to have “conventional” mechanisms of failure including infection (3 patients), impingement due to malpositioning (3 patients), fracture (1 patient, who was referred with unexplained pain and sustained a fracture secondary to minor trauma after the first outpatient visit), and primary loss of acetabular fixation (1 patient). Of the remaining patients, 14 of 31 had no intraoperative features apart from synovitis. A significant soft tissue mass was determined in 10 of these 31 patients (1 further patient who was found to be infected also had a soft tissue mass); 4 of the 10 had a solid pseudotumor (Figure 5) and the remainder were fluid-filled. In 7 patients, there was significant acetabular osteolysis but no soft tissue mass (Figure 4).

**Table 3.** Types of failure

	Imaging	Surgical	Hazards	Surgical plan
Type I:				
Conventional failure modes	Normal or small fluid-filled ‘pseudotumor’ on MRI	Variable	Misdiagnosis, Recurrent infection	
Type II:				
Synovitis with negative investigations	Normal or small fluid-filled ‘pseudotumor’ on MRI	Varying degrees of synovitis	Misdiagnosis	Need to exclude other causes e.g. infection, mechanical causes. Consider further imaging for psoas, frozen section during revision
Type III:				
Soft tissue disruption	MRI shows fluid or solid mass with variable soft tissue and muscle destruction (Figures 2 and 3)	Abductors may be atrophic or avulsed/absent	Instability after revision	Plan for possibility of muscle loss, including need for musle reconstruction (e.g. graft jacket) or captive cup. Like type V, may need pelvic surgeon for full excision of intrapelvic mass
Type IV:				
Bone destruction	Osteolysis on CT/plain films. MRI as type I (Figure 4)	Loose cup. Soft tissue reaction varies	Loss of bone stock and need for extensive reconstruction	May need extensive reconstruction. CT and pelvic surgeon may be helpful. Early surgery indicated to prevent fracture
Type V:				
Solid pseudotumor	MRI shows large mass. Mass may extend to pelvis (Figure 5)	Massive soft tissue reaction but musculature may be intact	Secondary infection if incompletely excised	Complete excision required. May need pelvic exploration

**Figure 2.**
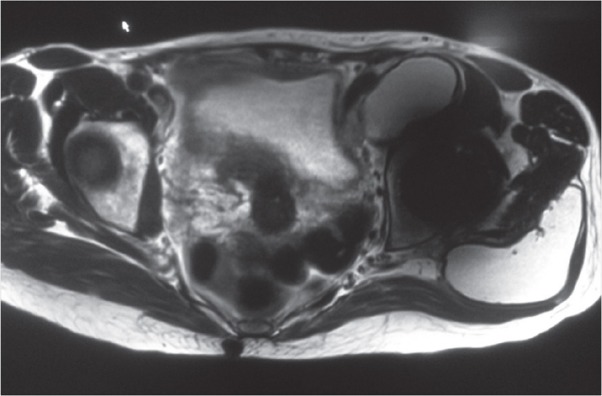
MRI of case 30, showing large fluid-filled pseudotumor.

**Figure 3.**
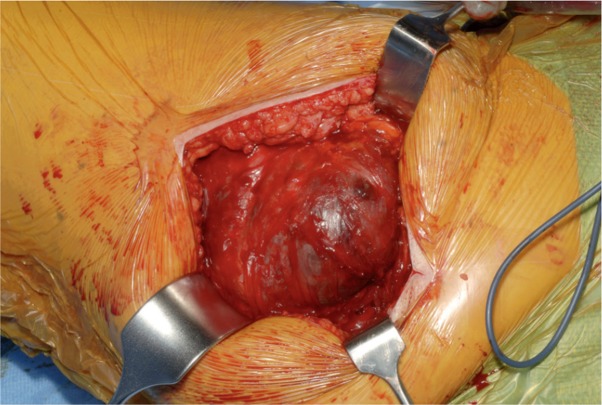
Intraoperative image of case 30.

**Figure 4.**
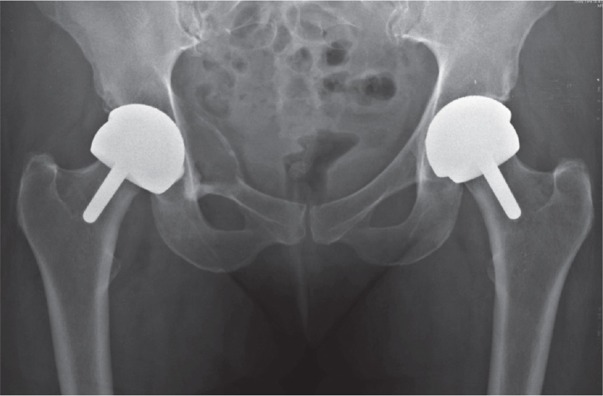
Preoperative radiograph of patient 34 demonstrating extensive osteolysis adjacent to the right acetabular component.

**Figure 5.**
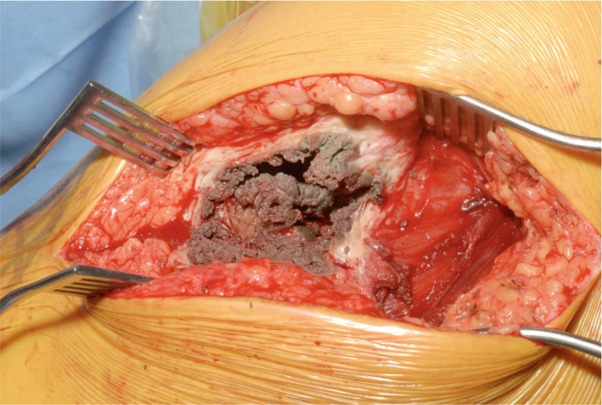
Case 32 with destructive solid pseudotumor.

### Correlation between MRI and operative findings

The presence of a mass on MRI was predictive of a mass at operation (chi-square test, p = 0.01). MRI frequently showed a small, fluid-filled pseudotumor that was not felt to be significant intraoperatively. As a result, in this cohort MRI had a sensitivity of 85% and a specificity of 59% for the presence of a mass observed intraoperatively.

### Surgery performed (Table 2)

In most cases, primary THR stems were used with metal-on-polyethylene or ceramic-on-ceramic bearings. 2 patients underwent their revision surgery early in this series to stemmed MOM arthroplasty with retention of the acetabular component, but as a result of the findings reported in this study this is no longer our practice. In cases of severe pelvic osteolysis, pelvic reconstruction was necessary. Captive cups were used in 2 hips (1 patient) with extensive muscle loss.

### Analysis of retrieved components

Of the hips that failed by non-conventional means, 14 of 31 were determined to have failed with high material loss and then high levels of metal ions. The median levels of metal ions in this group were 40 (10–166) ppb for cobalt and 32 (3.5–117) ppb for chromium. Median wear rates were 20 (4.2–84) µm/year for the head and 7.0 (2.0–60) µm/year for the cup. All cases had inclination, version, or both outside the range described as acceptable by Grammatopoulos et al. (2010). The incidences of pseudotumor (9/14 vs. 9/17, p = 0.9) and of muscle or bone destruction (3/14 vs. 3/17, p = 0.6) were no different in this group than in the study population as a whole; there was no statistically significant difference in outcome between the 2 groups (Mann-Whitney U test: median ΔOHS 22 (6–35) in the wear group and 15 (4–24) in the non-wear group; p = 0.09). The remaining 19 patients with no conventional mechanism of failure showed no sign of material loss (with normal or near-normal metal ion levels and a normal wear pattern on retrieval analysis). Analysis of the patients who were found to have conventional mechanisms of failure revealed excess wear and high metal ion levels in 2 of 8 patients, both of whom were found to be infected.

### Outcomes and complications (Table 4)

The median OHS was 37 (9–48) at the latest follow-up, a median change of OHS (ΔOHS) of +17 points (–10 to 41) when compared to the preoperative score (Wilcoxon signed rank test, p < 0.001). Although the number of patients involved was small, the 2 patients who underwent femoral component-only revision had a statistically significantly poorer ΔOHS than those who underwent revision of both bearing surfaces (n = 37). Patients with a femoral component-only revision with retention of a MOM articulation had a median OHS of 27 at 3 years, with a median ΔOHS of –3, as compared to a median OHS of 37 with a median ΔOHS of +19 (at a median follow-up time of 19 months, p = 0.02). Patients with a higher preoperative Co level had a greater ΔOHS, although this was not the case for Cr (Spearman’s correlation coefficient was 0.40 for Co (p = 0.02) and 0.21 for Cr (p = 0.2)).

**Table 4.** Outcomes

Type	Description	n	Preoperative OHS median (range)	Postoperative OHS median (range)	ΔOHS (range)	p-value
Whole cohort		39	15 (2–31)	36.5 (9–48)	17 (–10 to 41)	< 0.001
Subgroup						
1	Conventional	8	17 (2–30)	32 (20–46)	17 (–10 to 41)	0.08
2	Synovitis	14	11 (4–29)	33 (9–44)	14 (4–33)	0.002
3	Soft tissue disruption	6	27 (14–31)	47 (29–48)	16 (6–33)	0.04
4	Bone destruction	7	20 (10–28)	41 (39–45)	22 (12–35)	0.03
5	Solid pseudotumor	4	8 (2–22)	29.5 (15–31)	16.5 (9–23)	0.07

Outcome was similar in patients with failure by conventional mechanisms and in the remainder of the group (Table 4).

8 of the 36 patients who underwent MRI did not present with either a mass on MRI or with cobalt or chromium above the MHRA-recommended maximum of 7 ppb (3 other patients had normal metal ions but did not undergo MRI; they have been excluded from this analysis). Median latest OHS was 31 (19–46) in this group, as compared to 37 (9–48) in the group with at least one abnormal investigation, but this difference did not reach significance (median of differences = 7, CI: –4 to 15; p = 0.2). Similarly, ΔOHS appeared worse in the group with normal investigations (median 15 (4–24) vs. 18 (5–35) in patients with at least one abnormal investigation; median of differences = 5, CI: –2 to 14), but this was not statistically significant (p = 0.2).

There were 2 reoperations. 1 patient (who had a solid pseudotumor and massive destruction) had recurrent dislocations and underwent a revision of the acetabular component to a captive cup. 1 patient underwent a re-exploration for recurrent solid pseudotumor; at reoperation, the acetabular component was loose and it was revised. No patients developed a secondary infection but 1 patient (2 hips) died from a cause unrelated to the surgery.

## Discussion

This paper outlines our experience of revision of MOMHA, gained over a 4-year period in a tertiary referral unit, with a median follow-up time of 30 months. Due to the nature of the unit’s practice, the study population shows great heterogeneity of demographics, implant type, and manufacturer, and as a result we have encountered a wide variety of failure mechanisms. This has provided a challenge, which has been addressed using a multidisciplinary approach to preoperative planning, revision surgery, and postoperative follow-up. As the revision burden of this type of implant increases, these challenges are being encountered by more surgeons and more units. It is our intention that the lessons we have learned will be of help to units with less experience.

Overall, outcomes of revision surgery were acceptable with an overall median improvement in OHS of 17 points and with only 2 re-revisions. There was no difference in postoperative functional scores between patients revised for conventional failure mechanisms and those for mechanisms directly attributable to the MOM bearing. There were 2 groups of patients who had a poorer outcome. Firstly, patients who were revised to a stemmed MOM prosthesis had poorer results than those revised to other bearing types. On this basis, we do not recommend retaining a MOM articulation, regardless of the reason for revision. Secondly, 2 of 4 hips with solid pseudotumors have since undergone reoperation. These patients are particularly challenging and the presence of a solid pseudotumor at preoperative MRI is a significant finding. Surgeons should be aware of the difficulties posed by this group of patients, and the involvement of plastic surgeons or those with pelvic expertise may be helpful.

There was great variation in the mechanism of failure. A striking proportion of patients (8 of the 39 patients, all in patients referred with unexplained pain) were found to have conventional mechanisms of failure. Of those whose failure was directly attributable to the MOM articulation, only 14 showed component malpositioning with subsequent high wear rates and high blood metal ion levels. The remaining 17 patients had normal or near-normal metal ion levels and acceptable component positioning. The proportions of patients with a pseudotumor and overall outcomes were similar in each group.

There was also great variation in the preoperative and intraoperative findings encountered. We have divided patients into 5 broad groups on the basis of preoperative and intraoperative findings, which we have found to be helpful in determining the amount of theater time required, the ordering of additional equipment for reconstruction, and the need for pelvic, vascular, or plastic surgical assistance with revision surgery (Table 3). In particular, each group has different requirements in terms of surgical technique and each presents particular challenges.

### Misdiagnosis

8 of 39 hips (all in patients referred with a diagnosis of unexplained pain), turned out to have conventional mechanisms of failure such as infection or impingement. A further 14 patients presented with pain in the absence of any significant soft tissue lesion or osteolysis—either on MRI or intraoperatively. Intraoperatively, the only finding was synovitis, but histological examination revealed appearances suggestive of a reaction to metal debris. Caution must be exercised in such cases, and there should be a low threshold for 2-stage surgery if infection is suspected. Particular care should be taken with patients with low metal ion levels and a normal MRI. The outcomes for this group of patients appeared to be worse overall, although this did not reach statistical significance and some patients had a favorable outcome. We do not consider normal investigations to be an absolute contraindication to revision surgery, but we now use diagnostic hip aspiration and injection of local anesthetic in all such patients and our threshold for revision has risen.

### Osteolysis

7 hips showed significant osteolysis at preoperative CT and intraoperatively. In 2 cases, significant pelvic reconstruction was performed with trabecular metal augments and posterior column plating for incipient pelvic discontinuity. The osteolysis observed in these cases may be due to the activation of osteoclasts secondary to the release of proinflammatory cytokines by osteoblasts exposed to high local levels of cobalt and chromium ions (Mabilleau et al. 2008), but over-reaming of the acetabulum and stress-shielding by rigid acetabular components may have played a role. All patients considered for surgery in our unit now undergo preoperative CT to assess acetabular orientation and the degree of osteolysis. We recommend that the equipment and expertise for pelvic reconstruction should be available for all cases, and that surveillance of asymptomatic MOMHA should include at least plain radiographs.

### Incomplete resection of pseudotumor and damage to surrounding structures

1 patient (1 hip) required reoperation for incompletely resected pelvic pseudotumor. Complete resection of pseudotumors is important to avoid recurrence and prevent secondary infection. MARS MRI is essential for preoperative planning in these cases, and can reveal the presence of a solid pseudotumor, intrapelvic extension requiring pelvic exploration, or involvement of other structures such as nerves and vessels. In 1 case, the pseudotumor entirely encased the sciatic nerve and dissection of the pseudotumor was performed with the aid of intraoperative nerve monitoring.

### Muscle destruction and instability

Previous series have shown a high incidence of dislocation following revision of MOMHA. Preoperative MRI is helpful to determine the presence of a large mass and muscle involvement, but these patients are challenging and require significant preoperative preparation. In 1 case, we have had to re-revise a patient with recurrent dislocation and in 2 cases, captive cups were necessary in the presence of significant soft tissue destruction.

Joint guidelines for the management of MOMHA from the European Hip Society and the European Federation of National Associations of Orthopaedics and Traumatology (EFORT) concur with earlier guidelines from the UK MHRA in suggesting close follow-up of large-diameter MOM bearings for the lifetime of the implant, with plain radiography and determination of metal ion levels (EFORT 2012, MHRA 2010). They recommend that cross-sectional imaging should be reserved for symptomatic patients or those with elevated metal ion levels, although the maximum safe level remains debatable (an issue reflected in the large number of symptomatic patients in this series presenting with one or more ion level below the 7 ppb suggested as a cutoff by the MHRA). Our findings support the existing guidelines. In addition, the following further conclusions can be drawn:

Preoperative planning with MARS MRI is useful for prediction of muscle destruction and the extent of any solid pseudotumor in a manner with which surgeons are familiar (communication between radiologist and surgeon is more straightforward with MRI images than with ultrasound snapshots). Routine involvement of a radiologist with experience of these cases can provide valuable information for preoperative planning. Patients with solid pseudotumors and/or extensive muscle or bone destruction may benefit from undergoing surgery in units with experience of this group of cases.

Surgeons should be aware of the possibility of severe pelvic osteolysis in MOM patients and plan for such an eventuality.

Raised blood metal ion levels, while predictive of high material loss, are not predictive of the degree of soft tissue or bone damage found at revision.

Caution should be exercised when revising patients with normal MRI and blood metal ion measurements. Such patients can have a good outcome, but carry a significant risk of misdiagnosis and should be investigated fully to exclude conventional mechanisms of failure.

Revision of MOMHA for any reason should include both sides of the bearing surface and result in a non-MOM bearing couple.
